# Restoring adenosine balance in axial spondyloarthritis: a stage-specific framework for immune and structural modulation

**DOI:** 10.3389/fimmu.2026.1738120

**Published:** 2026-03-10

**Authors:** Fataneh Tavasolian, Behdad Ravarian, Melissa Lim, Robert D. Inman

**Affiliations:** 1 Schroeder Arthritis Institute, Toronto Western Hospital, University Health Network, Toronto, ON, Canada; 2 Krembil Research Institute, University Health Network, Toronto, ON, Canada; 3 Hand Program, Division of Plastic, Reconstructive and Aesthetic Surgery, Toronto Western Hospital, University Health Network, Department of Surgery, affiliated with Temerty Faculty of Medicine, University of Toronto, Toronto, ON, Canada; 4 Division of Orthopedics, Osteoarthritis Research Program, Schroeder Arthritis Institute, University Health Network, Toronto, ON, Canada; 5 Spondylitis Program, Division of Rheumatology, Schroeder Arthritis Institute, University Health Network, Toronto, ON, Canada; 6 Departments of Medicine and Immunology, University of Toronto, Toronto, ON, Canada

**Keywords:** adenosin, axial spondyloarthritis, CD39, CD73, exosomes

## Abstract

Axial Spondyloarthritis (AS) is a chronic immune-mediated disease of the axial skeleton characterized by persistent inflammation and pathological bone formation driven by reciprocal signaling between immune and stromal cells. Central to this interplay is adenosine—a key metabolic regulator of immune tolerance and tissue remodeling. In AS, purinergic homeostasis is profoundly disrupted: the ectonucleotidases CD39 and CD73, responsible for adenosine synthesis, are downregulated, while adenosine-degrading enzymes ADA and its surface anchor CD26 are upregulated. This enzymatic disequilibrium depletes adenosine in inflamed tissues, impairs FOXP3^+^ regulatory T cell induction, and amplifies Th17-driven inflammation and fibroblast activation. We propose a stage-specific therapeutic framework for restoring adenosine balance in AS encompassing: (1) reconstitution of CD39/CD73 enzymatic activity, (2) receptor-selective modulation of A_2_A and A_2_B signaling pathways, and (3) exosome-mediated delivery of adenosine-regulating enzymes and microRNAs to reestablish immune homeostasis with cellular precision. The dual nature of adenosine—anti-inflammatory through A_2_A receptor activation and pro-fibrotic via A_2_B receptor engagement—necessitates context-aware targeting to suppress immune dysregulation without promoting ossification. This synthesis integrates molecular, cellular, and translational insights into a unified model of AS pathogenesis. By aligning mechanistic disruption with stage-specific and exosome-enabled interventions, it establishes a conceptual foundation for precision therapies aimed at recalibrating immune–stromal interactions and halting structural progression. This review synthesizes published mechanistic and translational evidence and includes hypothesis-generating therapeutic concepts that remain to be formally validated in AS.

## Introduction

Axial Spondyloarthritis (AS) is a chronic immune-mediated disease of the axial skeleton, characterized by enthesitis, inflammation, and progressive structural remodeling (1). Its pathogenesis is driven by a tightly interconnected network of immune and stromal cells whose dysregulated interactions sustain inflammation and promote pathological bone formation (2). The initiating events likely occur at mechanically stressed entheses, where microdamage and local cytokine release activate resident immune cells. Monocyte-derived macrophages and dendritic cells respond by producing IL-23, IL-6, and TNF-α, reshaping the local immune landscape. Dendritic cells not only polarize naïve T cells toward a Th17 phenotype, but also shape the cytokine gradient and influence stromal activation. Macrophages amplify the inflammatory loop through sustained cytokine production and altered receptor expression. Th17 cells, once expanded, become dominant drivers of inflammation, producing IL-17 and IL-22, which recruit neutrophils, activate stromal cells, and perpetuate tissue damage. Their resistance to suppression is reinforced by surface molecules that modulate enzymatic activity and cytokine sensitivity (3–5). FOXP3^+^ regulatory T cells (Tregs), which normally counterbalance Th17 responses, are numerically and functionally impaired in AS. Their failure to suppress Th17 expansion reflects a deeper disruption in the molecular cues that govern immune tolerance. The imbalance between Th17 and Treg subsets evolves as macrophages and dendritic cells continue to shape the cytokine environment, reinforcing Th17 dominance and suppressing resolution mechanisms (6–8).

**Figure 1:**
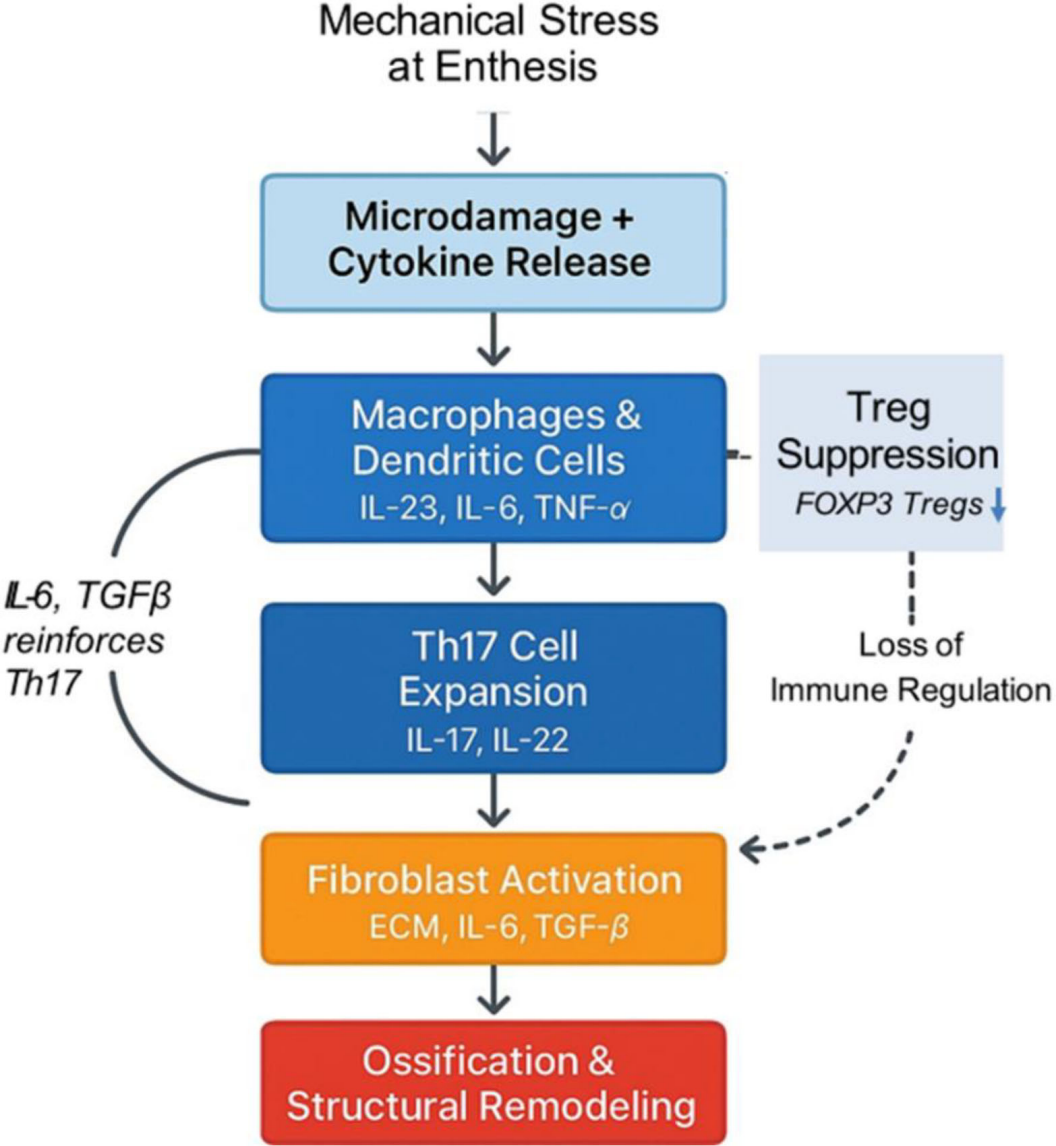
Cascade of immune–stromal interactions driving inflammation and ossification in AS. This schematic diagram illustrates the stage-specific cellular and cytokine network initiated by mechanical stress at the enthesis. Microdamage and local cytokine release activate monocyte-derived macrophages and dendritic cells, which secrete IL-23, IL-6, and TNF-α, reshaping the local immune environment. These signals promote the expansion of Th17 cells, which produce IL-17 and IL-22, driving neutrophil recruitment and fibroblast activation. Activated fibroblasts secrete ECM components, IL-6, and TGF-β, reinforcing Th17 polarization and inhibiting FOXP3^+^ Treg induction. This reciprocal loop between Th17 cells and fibroblasts sustains inflammation and initiates stromal remodeling. The loss of Treg-mediated suppression further amplifies immune dysregulation. Over time, fibroblast-derived signals activate Wnt, BMP, and TGF-β pathways in osteoprogenitor cells, culminating in pathological ossification and syndesmophyte formation. The diagram captures the self-reinforcing nature of immune–stromal crosstalk in AS and highlights key molecular checkpoints that may serve as therapeutic targets.

This immune dysfunction directly influences stromal behavior. Fibroblasts at the enthesis and periosteal regions respond to chronic cytokine exposure by shifting phenotype. They produce extracellular matrix components, remodeling enzymes, and osteogenic mediators. Critically, fibroblasts also release IL-6 and TGF-β, which reinforce Th17 polarization and inhibit Treg induction, creating a cytokine loop that links inflammation to ossification. In addition to soluble mediators, fibroblasts express adhesion molecules and chemokines that retain and reprogram immune cells locally, deepening the inflammatory niche. Their interaction with immune cells is reciprocal: fibroblasts recruit and shape immune cell differentiation, while immune cells sustain fibroblast activation (5, 9, 10). As fibroblasts activate Wnt, BMP, and TGF-β pathways, they influence nearby osteoprogenitor cells, which initiate new bone formation. This process leads to syndesmophyte development and spinal ankylosis—hallmarks of AS progression. The ossification is not a downstream consequence of inflammation but a parallel, tightly coupled pathway driven by cellular cross-talk (11–13). This cellular network—macrophages, dendritic cells, Th17 cells, Tregs, fibroblasts, and osteoprogenitors—operates as a dynamic system. Each node influences the others through cytokines, surface enzymes, receptor signaling, and spatial proximity. In AS, this system becomes self-reinforcing: immune dysregulation promotes stromal remodeling, which in turn sustains immune activation. The result is a persistent loop of inflammation and pathological bone formation, where immune and structural compartments are no longer distinct but entangled in a shared trajectory (**Figure 1**) (5, 12). Beneath this intricate cellular choreography lies a molecular conductor—adenosine. As a key regulator of immune and stromal interactions, adenosine weaves through the AS network, shaping inflammation, tolerance, and tissue remodeling. The next section explores how adenosine orchestrates these processes across cell types, setting the stage for understanding its disruption in disease (14). Throughout this review, we distinguish AS-direct observations from inferred mechanisms extrapolated from related autoimmune or stromal systems. Where therapeutic implications are proposed, they should be viewed as testable hypotheses rather than established clinical guidance in AS.

## Adenosine signaling in Ankylosing Spondylitis: a molecular cascade and intercellular network

Within the inflammatory microenvironment of AS, a silent but powerful regulatory system operates beneath the surface: purinergic signaling. This system, centered on the metabolism and receptor-mediated effects of adenosine, orchestrates a dynamic interplay between immune and stromal cells. In health, adenosine serves as a molecular brake, converting danger signals into immunosuppressive cues. In AS, however, this cascade is disrupted, leading to a breakdown in immune tolerance and a shift toward chronic inflammation and pathological tissue remodeling (15, 16). The cascade begins with the release of extracellular ATP, a potent danger-associated molecular pattern, from stressed or damaged cells at mechanically strained entheses. ATP and its derivative ADP activate P2 purinergic receptors on immune cells, triggering pro-inflammatory responses including the release of IL-1β, IL-6, and TNF-α. To prevent excessive inflammation, extracellular ATP must be rapidly degraded. This is achieved through a two-step enzymatic process: CD39 (ENTPD1), expressed on Tregs, monocyte-derived macrophages, and dendritic cells, hydrolyzes ATP and ADP into AMP. Subsequently, CD73 (NT5E), found on Tregs, mesenchymal stem cells (MSCs), and fibroblasts, converts AMP into adenosine (17–19). In AS, this enzymatic axis is compromised. Monocyte-derived macrophages from patients exhibit reduced expression of CD39, alongside decreased levels of A1 and A2B adenosine receptors, while A2A receptor expression is paradoxically increased. This imbalance contributes to impaired adenosine synthesis and altered receptor signaling, with elevated A2A expression failing to compensate for the enzymatic deficit. These changes correlate with disease severity and reflect a dysfunctional purinergic regulatory environment (20). CD39 and CD73 expression in peripheral blood mononuclear cells is diminished in untreated AS patients, correlating with impaired FOXP3^+^ regulatory T cell induction and enhanced Th17 polarization. These findings suggest that the initial steps of adenosine production are critically impaired in AS, undermining the immunosuppressive potential of this pathway (21). Simultaneously, adenosine degradation is accelerated. Adenosine deaminase (ADA), which converts adenosine into the inactive metabolite inosine, is elevated in the serum of patients with AS, particularly during active disease phases (22). ADA is anchored to the cell surface by CD26 (dipeptidyl peptidase-4), a molecule upregulated on Th17 cells and fibroblasts in AS. This anchoring facilitates pericellular adenosine clearance, effectively depleting the local microenvironment of adenosine and limiting its engagement with regulatory receptors (23).

Although the cause of reduced CD39 and CD73 expression in AS is not fully elucidated, several lines of evidence point to upstream inflammatory and metabolic pressures. Chronic exposure to IL-6, IL-17, and TNF-α—cytokines elevated in AS—has been shown in multiple autoimmune settings to repress transcription of ENTPD1 (CD39) and NT5E (CD73) on regulatory and antigen-presenting cells. In addition, hypoxia and metabolic stress at the enthesis may alter AMPK- and HIF-1α-dependent pathways that normally maintain ectonucleotidase expression. Finally, Th17-skewing transcriptional programs, particularly sustained STAT3 activation, are known to suppress CD39 in T cells and destabilize Treg lineage stability. Together, these mechanisms provide a plausible cytokine- and microenvironment-driven basis for ectonucleotidase downregulation in AS, although direct causal studies in AS remain needed (24–30).

Once generated, adenosine interacts with four G protein–coupled receptors—A1, A2A, A2B, and A3—each exhibiting distinct affinities and downstream signaling profiles. The A2A receptor (A2AR), with high affinity for adenosine, is expressed on macrophages, dendritic cells, Tregs, and CD4^+^ T cells. Its activation suppresses NF-κB and STAT3 signaling, reduces IL-6, TNF-α, and IL-17 production, and promotes FOXP3^+^ Treg differentiation (31). In AS, A2AR expression is paradoxically upregulated, particularly in macrophages, yet its immunosuppressive function remains underutilized due to insufficient adenosine availability (20). In contrast, the A2B receptor (A2BR) requires higher concentrations of adenosine for activation and is predominantly expressed on fibroblasts and osteoprogenitor cells. A2BR signaling activates Smad3, STAT3, and ERK1/2 pathways, leading to collagen synthesis, connective tissue growth factor (CTGF) expression, and extracellular matrix deposition (32). While A2BR is downregulated in AS immune cells, its expression is preserved in fibroblasts, suggesting a potential shift in adenosine signaling from immune regulation to stromal activation. This receptor’s role in promoting fibrosis and ossification in AS is supported by *in vitro* studies but remains to be validated *in vivo (*20). The A3 receptor (A3R), expressed on lymphocytes including Th17 cells, has demonstrated anti-inflammatory effects in other autoimmune contexts by suppressing IL-6, TNF-α, and NF-κB signaling (33). Its specific role in AS is not yet fully characterized, and current understanding is based on extrapolation from related diseases. Similarly, the A1 receptor (A1R), which modulates neutrophil chemotaxis and oxidative stress, is downregulated in AS macrophages. This reduction may contribute to excessive neutrophil recruitment and tissue damage at inflamed entheses, although direct evidence in AS is limited (20). Here explained the consequences of adenosine signaling disruption cascade through the cellular network. Macrophages, deprived of adenosine, fail to engage A2AR-mediated suppression, maintaining high levels of TNF-α and IL-6. Dendritic cells, lacking adenosine cues, continue to polarize naïve T cells toward the Th17 lineage. Th17 cells, protected from suppression by elevated CD26 and ADA, expand unchecked, producing IL-17 and IL-22, which activate fibroblasts and recruit neutrophils. Tregs, without adenosine support, lose their suppressive function and fail to restore immune tolerance. Fibroblasts, exposed to chronic cytokine stimulation and elevated adenosine in localized niches, activate A2BR, initiating fibrotic remodeling and ossification. Osteoprogenitor cells, influenced by fibroblast-derived signals and A2BR activation, begin ectopic bone formation, driving syndesmophyte development (11, 20, 23, 34–36). Beyond direct receptor signaling, adenosine intersects with several key intracellular pathways. A2AR activation inhibits NF-κB and STAT3, both of which are central to the transcription of pro-inflammatory cytokines and Th17 differentiation (37). In AS, impaired A2AR signaling allows persistent NF-κB activity, sustaining IL-6 and TNF-α production. A2BR activation, conversely, enhances STAT3 and Smad3 phosphorylation, promoting fibroblast proliferation and ECM deposition. The TGF-β pathway, a known driver of fibrosis and osteogenesis, is synergistically activated by A2BR signaling, linking adenosine metabolism to structural progression (36, 38, 39). Additionally, Wnt and BMP pathways, upregulated in AS fibroblasts and osteoprogenitors, interact with A2BR signaling to promote pathological bone formation (11). Emerging evidence suggests that adenosine may also modulate JAK/STAT and mTOR pathways, influencing immune cell fate and stromal behavior. While these interactions are well-characterized in other autoimmune diseases, their direct relevance to AS remains a hypothesis requiring further investigation (40–43).

**Figure 2:**
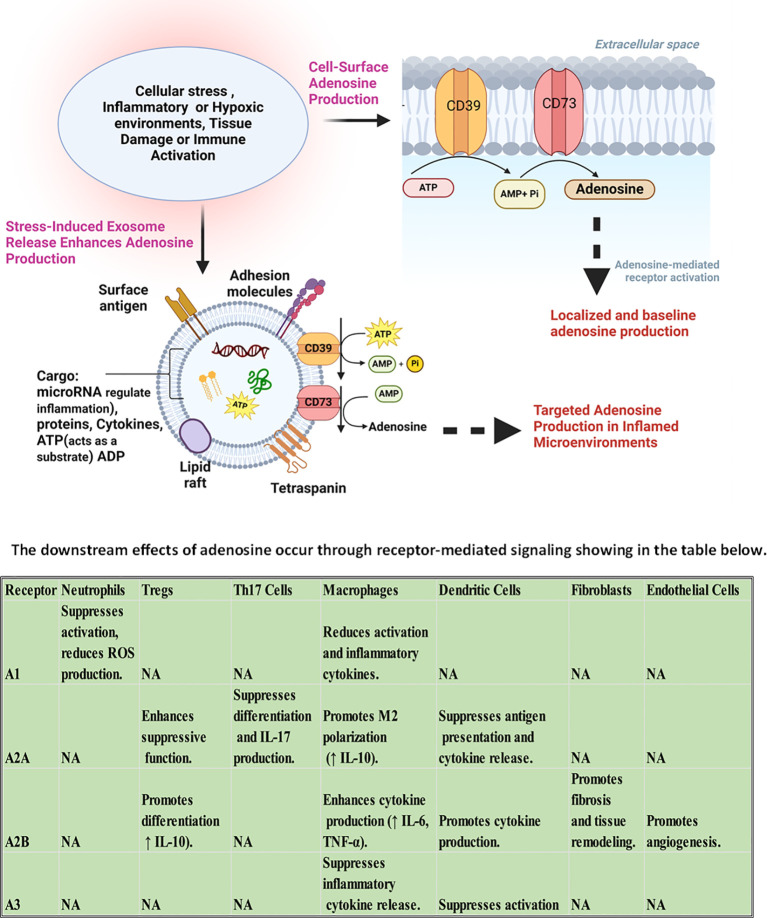
Adenosine production and receptor-mediated effects in inflammatory and hypoxic microenvironments. This schematic illustrates the dual pathways of adenosine generation and its downstream immunoregulatory and fibrotic effects in AS. Under conditions of cellular stress—including inflammation, hypoxia, tissue damage, or immune activation—extracellular ATP is released and sequentially hydrolyzed by the ectonucleotidases CD39 and CD73 into adenosine at the cell surface. In parallel, stress-induced exosome release contributes to localized adenosine production by delivering CD73, ATP/ADP substrates, and regulatory microRNAs; these vesicles act as mobile enzymatic platforms, amplifying adenosine synthesis in inflamed niches. The resulting adenosine engages four G protein–coupled receptors (A1, A2A, A2B, A3), each with distinct affinities and cell-specific effects. The accompanying table summarizes receptor-mediated outcomes across key immune and stromal cell types: A2A receptor activation promotes FOXP3^+^ Treg function, suppresses Th17 differentiation, and skews macrophages toward an anti-inflammatory M2 phenotype. In contrast, A2B receptor signaling—preferentially activated at higher adenosine concentrations—enhances fibroblast activation, promotes IL-6 and TNF-α production, and drives fibrotic remodeling and angiogenesis in stromal niches. A1 and A3 receptors can exert additional anti-inflammatory influences in specific contexts, including effects on neutrophils and antigen-presenting cells. Together, this figure highlights the spatial and functional complexity of adenosine signaling in AS and the rationale for receptor-selective and exosome-enabled strategies, “not directly established in AS” = effect reported in related systems but not yet shown directly in axial spondyloarthritis, † = context-dependent effect; outcome can vary with cell type, adenosine level, tissue environment, or disease stage.

In summary, adenosine signaling in AS is not a linear pathway but a multidimensional network. It connects immune suppression, fibroblast activation, and bone remodeling through a cascade of enzymatic reactions, receptor engagements, and intracellular signaling events. The disruption of this network—marked by impaired synthesis, accelerated degradation, and receptor misactivation—contributes to the persistent inflammation and structural damage characteristic of AS. Understanding this cascade in its full complexity offers a foundation for developing targeted therapies that restore immune balance while avoiding unintended activation of fibrotic mechanisms (**Figure 2**) (10, 16, 38, 43). The net effect of A_1_/A_2_A/A_2_B/A_3_ engagement is context-dependent, varying with cell type, adenosine concentration, disease stage, and tissue niche. Accordingly, receptor-selective strategies should be interpreted within these microenvironmental constraints and not as fixed binary outcomes.

## Experimental evidence supporting the adenosine disruption model

Disruption of adenosine signaling in AS is supported by human and preclinical studies that connect molecular changes to immunologic outputs. In monocyte-derived macrophages from AS patients, A_2_A receptor mRNA is increased, while CD39 (ENTPD1), A_1_ and A_2_B are decreased; A_2_A expression correlates inversely with BASFI, indicating that heightened receptor abundance does not compensate for insufficient enzymatic support to generate ligand (20). Consistent with a disease-specific signaling context, A_2_A activation increases IL-23 in AS macrophages and fails to suppress MMP8 (an effect seen in healthy cells), highlighting altered downstream responses in patient cells (44). At the systemic level, ADA2 activity is modestly elevated in AS serum (with total ADA and ADA1 not significantly different versus controls), supporting a bias toward accelerated extracellular adenosine degradation (22). Interventional evidence shows that the synthetic “brake” can be pharmacologically re-engaged: in a randomized clinical trial, nanocurcumin increased CD39 and CD73 expression, expanded FOXP3^+^ Tregs, and reduced Th17 polarization in AS peripheral blood, directly linking restoration of ectonucleotidase activity to improved T-cell balance (21). Complementing the human data, A_2_A agonism ameliorates disease in collagen-induced arthritis and CD73 is protective (CD73-deficient mice show worse CIA; a CD73-dependent A_2_A prodrug reduces inflammation), underscoring the causal role of adenosine generation and A_2_A signaling in restraining type-17–biased inflammation *in vivo (*45). By contrast, ADA inhibition with pentostatin worsened disease in rat CIA, cautioning that non-selective elevation of extracellular adenosine or global ADA blockade may have model- and context-dependent effects (46). Finally, multiple lines of evidence associate A_2_B signaling with fibrotic remodeling in lung/skin settings and dermal fibrosis (benefit with A_2_B antagonism), while other tissues (e.g., heart) report anti-fibrotic A_2_B effects; thus, A_2_B consequences are tissue-specific and must be interpreted within the AS lesion microenvironment (47). Taken together, human AS macrophage and serum data support a synthetic deficit (CD39 downregulated) combined with selective degradation bias (ADA2 upregulated), alongside context-specific receptor signaling (A_2_A under-liganded and IL-23-promoting in AS macrophages, A_2_B variably pro-fibrotic across tissues). This framework explains why simply increasing receptor expression or relying on indirect adenosine release (e.g., methotrexate mechanisms) may be insufficient in AS without restoring CD39/CD73-dependent synthesis and controlling pericellular degradation (**Tables 1**, **2**) (20).

**Table 1:** Enzymatic dysregulation in AS.

Enzyme	Function	Expression in AS	Impact
CD39	Converts ATP/ADP to AMP	Downregulated on Tregs and macrophages	Reduced ATP breakdown; impaired adenosine synthesis
CD73	Converts AMP to adenosine	Downregulated on immune cells; compensatory expression on fibroblasts	Reduced AMP conversion; impaired adenosine synthesis
ADA	Degrades adenosine to inosine	Upregulated in serum during active disease	Accelerated adenosine degradation
CD26	Anchors ADA to cell surface; enhances degradation	Upregulated on Th17 cells and fibroblasts	Facilitates pericellular adenosine clearance

**Table 2:** Adenosine receptor roles and expression patterns in AS.

Receptor	Affinity	Expression in AS	Function
A1	High	Downregulated in macrophages	Modulates neutrophil chemotaxis and oxidative stress
A2A	High	Upregulated in macrophages; underutilized due to low adenosine	Suppresses NF-κB/STAT3; promotes FOXP3^+^ Treg differentiation
A2B	Low	Preserved in fibroblasts and osteoprogenitors	Activates Smad3/STAT3; promotes fibrosis and ossification
A3	Intermediate	Expressed on Th17 cells; role in AS unclear	Suppresses IL-6, TNF-α, NF-κB in other autoimmune diseases

## Fibroblast remodeling and adenosine signaling in Ankylosing Spondylitis

Fibroblasts at the enthesis and periosteal regions are active drivers of inflammation and ossification in AS. In response to chronic immune activation, they adopt a pro-inflammatory and pro-osteogenic phenotype characterized by increased production of extracellular matrix (ECM) components, remodeling enzymes, and osteogenic mediators, thereby linking inflammation to new bone formation. Crosstalk with Th17 cells and macrophages through cytokines such as IL-17, IL-22, TNF-α, and IL-6 activates canonical Wnt, BMP, and TGF-β pathways in fibroblasts. In turn, fibroblast-derived IL-6 and TGF-β reinforce Th17 polarization and inhibit FOXP3^+^ Treg induction, creating a reciprocal feedback loop that connects immune dysregulation to structural remodeling (10, 48–50). Adenosine signaling modulates fibroblast behavior within this cytokine network. While A_2_A receptor activation generally restrains inflammation, AS fibroblasts are more responsive to A_2_B signaling. The A_2_B receptor—predominantly expressed on fibroblasts and osteoprogenitor cells and requiring higher adenosine concentrations for activation—can be engaged in localized stromal niches where compensatory CD73 expression allows transient adenosine accumulation despite overall ADA/CD26-driven degradation. This receptor shift reflects a redistribution of adenosine signaling from immune regulation toward stromal activation (30, 51–54). Upon activation, A_2_B signaling triggers Smad3, STAT3, and ERK1/2 phosphorylation, up-regulates connective-tissue growth factor (CTGF), and increases collagen I and fibronectin synthesis—hallmarks of fibrotic remodeling. These pathways converge with Wnt and BMP/TGF-β signaling to amplify osteogenic differentiation and contribute to syndesmophyte formation. While these signaling effects are firmly established in dermal and pulmonary fibroblast models, enthesal fibroblasts and osteoblast precursors from AS patients exhibit similar *in-vitro* activation signatures—including elevated COL1A1 and CTGF expression—supporting the presence of conserved adenosine-responsive mechanisms in the AS microenvironment (32, 48, 55, 56). Immune-origin purinergic imbalance—characterized by reduced CD39 and CD73 expression on regulatory and innate immune cells, and increased CD26 (DPP4) expression on activated T cells, including Th17 cells, with reported upregulation on fibroblasts in inflamed or fibrotic tissues (though AS-specific fibroblast data remain limited)—contributes to dysregulated adenosine metabolism and signaling. reduces global adenosine availability yet redistributes signaling toward stromal microdomains where A_2_B receptors predominate. Fibroblasts therefore both sense and amplify purinergic dysfunction, translating immune cues into structural change and establishing a biochemical bridge between inflammation and ossification (10, 23, 48, 54). Therapeutically, enhancing A_2_A signaling may suppress inflammation, but non-selective adenosine elevation risks engaging A_2_B and driving fibrosis. Therefore, targeting adenosine metabolism and receptor engagement with spatial precision—for example, restoring CD39/CD73 activity in immune compartments while preventing excessive A_2_B stimulation in stromal niches—is essential to avoid inadvertent activation of ossification pathways (39, 57, 58).

**Figure 3:**
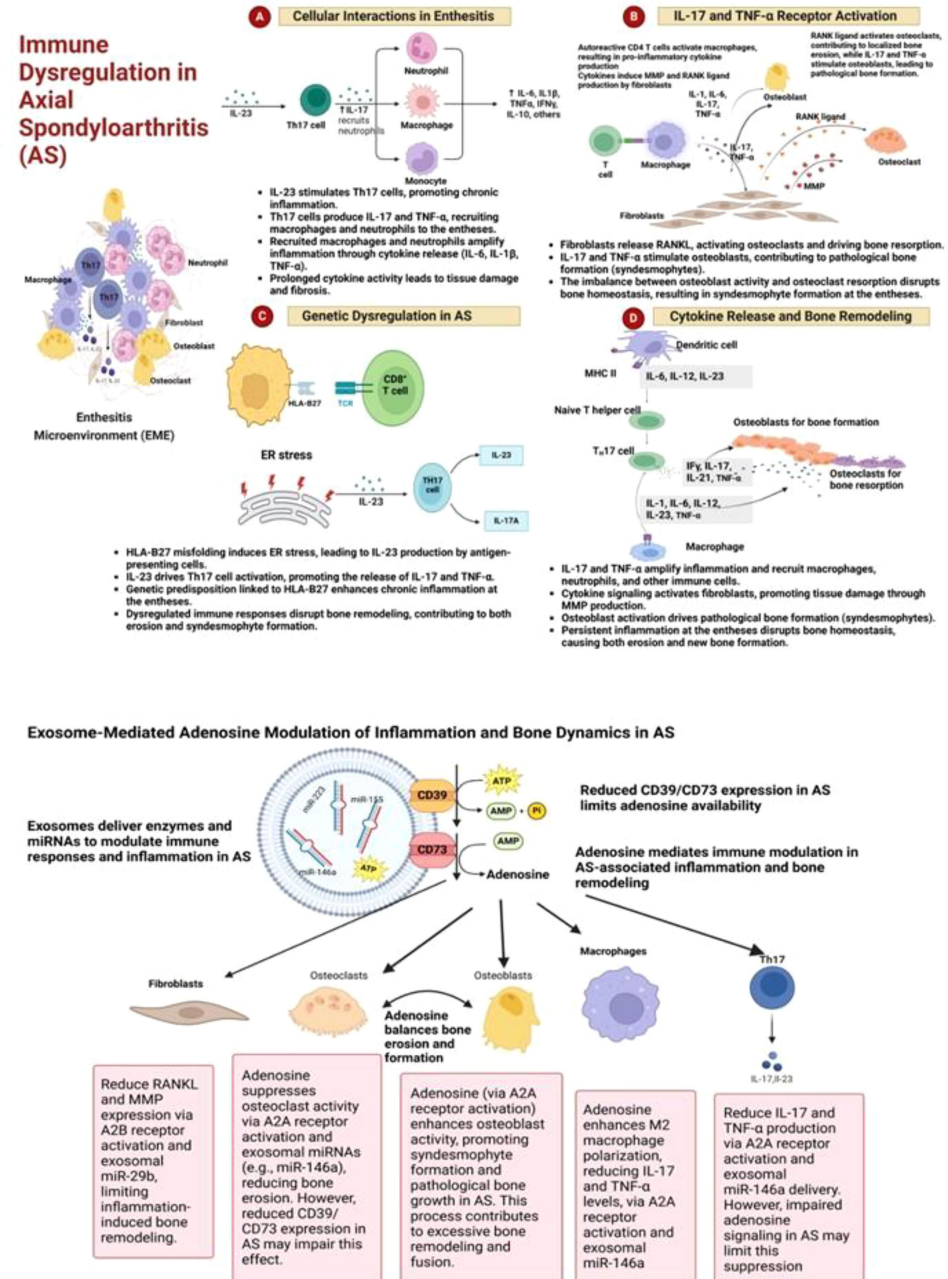
Integrated mechanisms of immune dysregulation, genetic predisposition, and adenosine-mediated modulation in AS. This composite figure illustrates the multifaceted pathogenesis of AS, integrating immune–stromal crosstalk, genetic drivers, cytokine signaling, and purinergic regulation. (1) Cellular interactions in enthesitis: Mechanical stress and microdamage at the enthesis activate macrophages and dendritic cells, which secrete IL-23, IL-6, and TNF-α. These cytokines promote Th17 cell expansion and IL-17/IL-22 production, which in turn activate fibroblasts. Activated fibroblasts release RANKL and pro-osteogenic mediators, driving osteoclastogenesis and initiating bone resorption. (2) Genetic dysregulation in AS: Misfolding of HLA-B27 in antigen-presenting cells induces endoplasmic reticulum (ER) stress, triggering IL-23 production and perpetuating Th17-driven inflammation. This genetic predisposition reinforces the chronic inflammatory loop at the enthesis. (3) Cytokine-driven bone remodeling: Persistent IL-17, IL-6, and TNF-α signaling promotes osteoclast differentiation and activity, while fibroblast-derived TGF-β and Wnt/BMP pathway activation stimulate osteoblasts and osteoprogenitor cells, leading to pathological new bone formation. (4) Exosome-mediated adenosine modulation: In healthy conditions, exosomes enriched with CD39 and CD73 catalyze the conversion of extracellular ATP to adenosine, which suppresses inflammation via A2A receptor activation on immune cells. In AS, reduced CD39/CD73 expression and elevated ADA/CD26 activity impair adenosine availability, weakening immunosuppressive signaling and allowing unchecked inflammation and stromal activation. Together, these interconnected pathways establish a self-reinforcing cycle of immune dysregulation, stromal remodeling, and ossification, highlighting multiple therapeutic entry points—including exosome-based adenosine restoration and receptor-selective modulation.

Collectively, these features position fibroblasts as central hubs that couple immune signals to structural progression within the disrupted adenosine network. These fibroblast-centered mechanisms provide the structural substrate upon which exosomal and cytokine-mediated regulation further act, as discussed in the following section (**Figure 3**) (58–60).

## Exosome biology and immune–stromal modulation in AS

Imagine a future clinical scenario: a patient with early-stage AS, showing signs of Th17 expansion and impaired Treg function, receives an infusion of engineered exosomes derived from autologous regulatory T cells. These vesicles are enriched with CD73 and miR-146a, designed to restore adenosine synthesis and suppress NF-κB signaling. Delivered intravenously, the exosomes home to inflamed immune compartments, where low ADA/CD26 activity allows adenosine accumulation and A_2_A receptor engagement. Within days, the patient’s peripheral blood shows increased FOXP3^+^ Tregs and reduced IL-17 levels—marking a shift toward immune resolution without triggering fibrosis (61).

Practical constraints on differential targeting. Notwithstanding this rationale, differential targeting of exosome cargo remains a major translational barrier. Systemically administered vesicles show preferential uptake by liver/spleen and stromal niches, making it challenging to restrict CD39/CD73 delivery to immune compartments while avoiding fibroblast-rich microenvironments where increased adenosine could activate A_2_B-driven fibrotic pathways. Ligand-modified exosomes, biomarker-guided patient stratification (e.g., CD39/CD73, ADA/CD26 activity), and immune-cell–specific targeting motifs will likely be required to achieve the necessary compartmental precision in AS (62–64). The above scenario is presented as a conceptual example to illustrate potential design principles rather than clinical evidence. Preclinical pharmacokinetic/biodistribution (PK/BD) and safety validation will be required prior to clinical translation in AS.

This hypothetical case illustrates the therapeutic potential of exosomes in AS, where vesicles act not only as biomarkers but as precision delivery vehicles for immunoregulatory cargo. Exosomes are nanosized extracellular vesicles (≈30–150 nm) released by immune and stromal cells. Their cargo—proteins, lipids, enzymes, and nucleic acids—reflects the activation state of the parent cell and enables targeted intercellular communication. In AS, plasma-derived exosomes show increased tetraspanin markers (CD63, CD81) and a pro-inflammatory shift in cytokine content, including reduced IL-10 and elevated IL-17. Functionally, AS plasma exosomes impair Treg proliferation and enhance IL-17 production in co-culture experiments—demonstrating their capacity to propagate disease-relevant programs (65). In contrast, many mechanistic insights into exosome cargo loading, uptake, and immune modulation are extrapolated from other autoimmune diseases. For example, Treg-derived exosomes have been shown in human studies to transfer miRNAs to dendritic cells, shifting them toward an IL-10–high, IL-6–low phenotype. Similarly, macrophage exosomes can shuttle cytokines and regulatory miRNAs that influence T-cell differentiation. These mechanisms, while not yet fully validated in AS, provide a conceptual scaffold for therapeutic design (66–68). Exosomes also intersect with the purinergic pathway. Immune- and mesenchymal-derived vesicles can carry CD73 (and in some contexts CD39), enabling local conversion of extracellular ATP/AMP to adenosine on the vesicle surface. In AS, where CD39/CD73 expression is reduced on immune cells and ADA/CD26 is elevated on Th17 cells and fibroblasts, vesicle-based adenosine production may offer a compensatory mechanism—if targeted delivery avoids stromal niches where A_2_B receptor activation could drive fibrosis (69–71). Exosomal microRNAs further modulate immune and fibrotic pathways. Anti-inflammatory miRNAs such as miR-146a and miR-29b suppress NF-κB and TGF-β signaling, while miR-21 and miR-155 promote fibroblast activation and extracellular matrix production. These polarity pairs mirror the A_2_A versus A_2_B split in adenosine signaling, with vesicle cargo composition helping determine whether local circuits resolve or progress (72–76). Therapeutically, exosomes must be engineered with stage-specific and compartment-aware precision. CD39/CD73-enriched vesicles may restore adenosine synthesis in immune compartments, while miRNA-loaded vesicles can suppress pro-fibrotic signaling. However, delivery must avoid fibroblast-rich niches with high ADA/CD26 activity to prevent unintended A_2_B engagement. Surface ligands targeting immune cells and biomarker-guided stratification (e.g., CD39/CD73 levels, ADA activity) may help achieve this selectivity (77, 78).

In sum, exosomes in AS are not passive bystanders but active participants in immune–stromal crosstalk. Their dual role—as amplifiers of inflammation and potential correctors of purinergic imbalance—makes them promising tools for both diagnostics and therapy, provided their design respects the spatial and molecular constraints of the AS microenvironment (65, 79–82).

## Balancing inflammation and fibrosis: a stage-specific approach to targeting AS

Adenosine signaling in AS functions as a dynamic, stage-dependent network shaped by the inflammatory microenvironment. In early disease, adenosine acts as a potent immunoregulatory molecule: by engaging high-affinity A_2_A receptors on T cells, macrophages, and dendritic cells, it suppresses NF-κB and STAT3 signaling, reduces IL-6, IL-17, and TNF-α production, and promotes FOXP3^+^ regulatory T-cell differentiation. These effects favor immune resolution and limit tissue injury when the pathway is activated promptly during initial inflammation (20, 83). As the disease advances, persistent ATP release from damaged tissues and compensatory CD73 expression on stromal cells can locally raise adenosine concentrations. In this altered milieu, low-affinity A_2_B receptors on fibroblasts and osteoprogenitor cells become engaged, triggering Smad3, ERK1/2, and STAT3 activation that drives fibroblast proliferation, collagen synthesis, and osteogenic differentiation. This receptor shift marks the transition from immune control to structural remodeling and syndesmophyte formation (10, 48). Therapeutic strategies must therefore align with disease stage. Early intervention should enhance A_2_A receptor engagement to suppress inflammation and restore tolerance, whereas chronic or fibrotic stages require avoidance of global adenosine elevation and may benefit from combined A_2_B antagonism or co-inhibition of downstream fibrotic cascades such as TGF-β, JAK/STAT, or mTOR. The key challenge is to restore immunoregulatory signaling without promoting fibrosis (38, 84). Exosome-based therapies illustrate this duality. CD39/CD73-enriched exosomes may reestablish adenosine production and support A_2_A-mediated suppression, but in fibroblast-rich niches with high ADA/CD26 activity they could also drive A_2_B-dependent fibrosis. Likewise, miR-146a- and miR-29b-containing exosomes can suppress NF-κB and TGF-β signaling, yet their net effect depends on disease stage and delivery site. Achieving benefit will require precise control of exosomal cargo and tissue targeting to confine activity to immune compartments while avoiding stromal activation (73, 85, 86). Biomarker-guided stratification offers a potential path forward. Quantifying CD39/CD73 expression, ADA activity, and receptor distribution across immune and stromal cells could inform patient selection and dosing. Delivery systems engineered with tissue-specific ligands might further restrict therapy to immune targets, minimizing structural risk (20).

Operationalizing this selectivity will require biodistribution control and cell-type–specific homing, which remain unresolved in current EV platforms.

In summary, adenosine’s role in AS evolves with disease progression. Therapeutic success will depend on restoring signaling with spatial and temporal precision—enhancing A_2_A-mediated suppression in early inflammation while preventing A_2_B-driven fibrosis in advanced stages.

## Integrated model of adenosine dysregulation and immune–stromal crosstalk in AS

**Figure 4:**
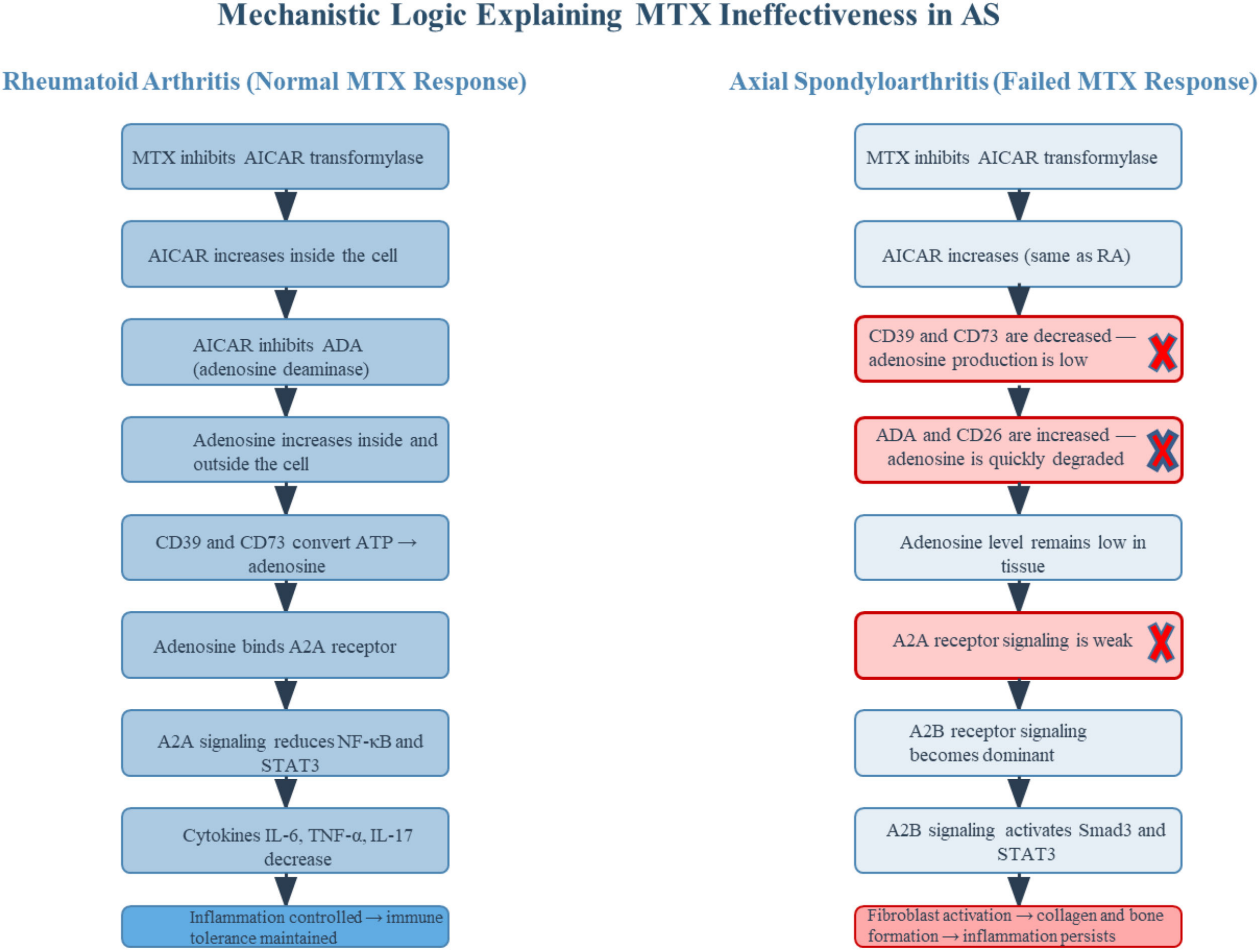
Mechanistic divergence explaining methotrexate (MTX) ineffectiveness in AS compared to RA. This comparative schematic illustrates a potential mechanism for MTX’s limited effect in AS but effective in RA and should be interpreted as a hypothesis pending AS−specific validation. Left panel (RA): MTX inhibits AICAR transformylase, leading to intracellular accumulation of AICAR, which inhibits ADA. This results in increased extracellular adenosine levels. Concurrently, CD39 and CD73 ectonucleotidases efficiently convert extracellular ATP to adenosine. The accumulated adenosine activates high-affinity A2A receptors on immune cells, suppressing NF-κB and STAT3 signaling pathways and reducing pro-inflammatory cytokines (IL-6, TNF-α, IL-17). This cascade promotes immune tolerance and clinical remission. Right panel (AS): Although MTX similarly increases AICAR, the downstream adenosine pathway is disrupted. CD39 and CD73 expression is reduced on immune cells, impairing adenosine synthesis. Simultaneously, ADA and its surface anchor CD26 are upregulated on Th17 cells and fibroblasts, accelerating adenosine degradation. As a result, extracellular adenosine remains insufficient to activate A2A receptors. Instead, localized adenosine accumulation in stromal niches engages low-affinity A2B receptors on fibroblasts and osteoprogenitors, triggering Smad3 and STAT3 signaling. This promotes collagen synthesis, connective tissue growth factor (CTGF) expression, and pathological bone formation. Thus, MTX frequently fails to suppress inflammation and may inadvertently contribute to fibrotic remodeling in AS. This figure encapsulates the concept of purinergic resistance in AS and underscores the need for stage-specific, receptor-selective, and compartment-aware therapeutic strategies that go beyond conventional MTX mechanisms. The AS panel represents a mechanistic hypothesis; the relative contributions of ectonucleotidase deficiency and ADA/CD26-mediated degradation to MTX non-responsiveness remain to be validated in AS.

Adenosine dysregulation in AS unfolds through a sequence of molecular and cellular disruptions that evolve across disease stages. The Three-Phase Adenosine Disruption Model integrates immune, stromal and therapeutic dimensions into a single framework, clarifying how adenosine imbalance drives disease progression, limits methotrexate (MTX) efficacy and reveals opportunities for phase-specific intervention (87) (**Figure 4**).

### Phase 1 — enzymatic deficiency and early immune activation

Microdamage at the enthesis activates macrophages and dendritic cells, releasing ATP and pro-inflammatory cytokines. In health, extracellular ATP is hydrolyzed to adenosine by CD39 and CD73, promoting FOXP3^+^ Treg differentiation and restraining Th17 expansion (88). In AS monocyte-derived macrophages, CD39 is downregulated (with A2A mRNA increased), whereas a reduction of CD73 is not established at the macrophage level. In peripheral blood, both CD39 and CD73 are reduced at baseline and can be restored by pharmacologic intervention (20). Net effect: adenosine synthesis is insufficient in immune compartments. This bottleneck diminishes A2A engagement and weakens suppression of NF-κB/STAT3, permitting sustained Th17 activity. Because MTX’s anti-inflammatory effect relies on an intact CD39/CD73 axis to elevate extracellular adenosine (as shown in RA), a deficient synthetic scaffold helps explain its limited impact in AS. Exosomes from Tregs (CD73^+^; CD39 variably reported) and MSC-EVs (commonly CD73^+^) could, in principle, supply ecto-enzymatic activity to partially compensate for this early deficit—an approach consistent with observed enzyme distributions but requiring dedicated AS testing (20, 21).

### Phase 2 — accelerated degradation and immune–stromal transition

With ongoing inflammation, stromal programs intensify. Even where compensatory CD73 on stromal cells transiently increases local adenosine, degradation is accelerated by ADA, presented at the surface via CD26 (DPP4) on activated T cells (including Th17) and reported on fibroblasts in inflamed/fibrotic tissues. Rapid conversion of adenosine to inosine limits A2A engagement, sustaining IL-17/IL-6/TGF-β loops that drive immune-to-stromal transition. In this context, modest MTX-driven adenosine increases are readily neutralized. Combining MTX with ADA inhibition or A2A agonism in AS remains untested; given mixed preclinical results for ADA blockade in arthritis, any such strategy should be considered hypothesis-level. A more physiologic concept is engineered exosomes designed to localize ecto-enzymes to immune niches or to minimize pericellular ADA access; however, these remain proposals requiring empirical validation in AS models (22, 23, 89, 90).

### Phase 3 — receptor misactivation and fibrotic progression

In advanced disease, fibroblast-driven tissue remodeling and ossification predominate. Local adenosine accumulation—whether due to persistent inflammation or therapy—can preferentially activate low-affinity A2B receptors expressed on fibroblasts and osteoprogenitors. In fibroblast systems, A2B signaling engages Smad3, STAT3 and ERK1/2, increases CTGF, and upregulates collagen I and fibronectin, consistent with fibrotic remodeling. AS *in vitro* studies in entheseal fibroblasts/osteogenic precursors show parallel activation signatures, while direct *in vivo* confirmation in AS remains limited. Thus, the purinergic axis can shift from immune regulation (A2A) toward stromal activation (A2B) in specific niches, aligning with structural progression (10, 48, 56, 91–95).

## Integrated interpretation and therapeutic outlook

Across these phases—impaired synthesis, accelerated degradation, receptor misactivation— AS may develop a purinergic resistance state that may help explain both progression and MTX non-responsiveness, and this remains to be validated in AS (96, 97). Therapeutic strategies should be phase-specific and biomarker-guided (CD39/CD73 levels, ADA/ADA2 activity, receptor distribution). In earlier disease, approaches that restore ectonucleotidase function or enhance A2A engagement—potentially with CD73^+^/Treg-exoxomes support—may recalibrate immune tolerance. In later stages, selective A2B antagonism and anti-fibrotic co-therapies (e.g., TGF-β/STAT pathway targeting) should avoid non-selective adenosine elevation that risks A2B-driven fibrosis. Current evidence is primarily *in vitro*/ex vivo for AS stromal biology, but the model is testable and provides a rational foundation for precision therapeutics that integrate purinergic and exosome biology (**Table 3**).

**Table 3:** Integrated three-phase model of adenosine disruption and therapeutic implications in AS.

Disease phase	Key pathological features	Dominant molecular disruptions	Impact on adenosine & MTX response	Therapeutic implications
Early Immune Phase	Enthesial microdamage; macrophage and DC activation; Th17 expansion; impaired Treg induction	↓ CD39/CD73 expression → impaired adenosine synthesis; ↑ extracellular ATP → P2 receptor activation	Low adenosine availability prevents A_2_A receptor engagement → MTX fails to amplify anti-inflammatory signaling	Restore CD39/CD73 function; A_2_A receptor agonism; P2 receptor blockade to reduce ATP-driven inflammation
Transitional Phase	Persistent inflammation; fibroblast activation; IL-6/IL-17/TGF-β cytokine loops	Compensatory CD73 on stroma; ↑ ADA/CD26 expression → accelerated adenosine degradation	Rapid clearance of adenosine → reduced A_2_A activation; MTX effect neutralized by ADA/CD26 activity	Inhibit ADA/CD26; modulate fibroblast–immune crosstalk; controlled A_2_A stimulation to restore immune tolerance
Late Fibrotic Phase	Fibroblast-driven ossification; extracellular matrix deposition; syndesmophyte formation	Local adenosine accumulation; predominant A_2_B receptor activation → Smad3/STAT3/ERK1/2 signaling	A_2_B-mediated fibroblast activation → collagen and CTGF induction; excess adenosine may worsen fibrosis	A_2_B receptor antagonism; anti-fibrotic co-therapy (e.g., TGF-β or JAK/STAT inhibitors); precision-targeted adenosine modulation

## Unresolved questions and opportunities for innovation in AS

The therapeutic landscape of AS is evolving, yet the purinergic axis remains largely unexplored. Current clinical management relies heavily on biologic agents targeting TNF-α and IL-17, which have demonstrated efficacy in reducing inflammation and improving functional outcomes. However, these therapies do not fully prevent structural progression, and a subset of patients remains refractory. Moreover, no existing treatments directly address the metabolic and enzymatic disruptions that underlie purinergic imbalance in AS (98, 99). This review has outlined how adenosine dysregulation drives immune imbalance and structural remodeling. Translating these mechanistic insights into clinical application requires addressing key gaps across molecular, cellular, and translational domains. Exosome-mediated restoration of CD39 and CD73 represents one of the most biologically aligned approaches to re-establishing adenosine balance. These vesicles can deliver functional enzymes, microRNAs, and regulatory proteins directly to immune compartments, offering a physiologic route to reinstate purinergic signaling where endogenous expression is lost (77, 78). Recent studies have demonstrated that CD39 and CD73 can be delivered via engineered exosomes or recombinant fusion proteins (RAIN), effectively restoring adenosine synthesis and suppressing inflammation in preclinical models of inflammatory disease (77). These findings support the feasibility of exosome-based purinergic modulation in AS and reinforce the need for compartment-specific targeting to avoid A_2_B-driven fibrosis. Beyond enzyme replacement, exosomes can be engineered to carry miRNAs that suppress ADA/CD26 or modulate A_2_A/A_2_B receptor expression. Although such designs remain conceptual, they position exosomes as adaptable tools for phase-specific adenosine recalibration (100). Other purinergic-based interventions are also emerging. Agents such as α,β-methylene ADP (APCP) and MEDI9447 (Oleclumab)—CD73 inhibitors originally developed for oncology—illustrate the druggability of this pathway but would require re-engineering for autoimmune use, since systemic CD73 inhibition could worsen inflammation. Similarly, CRISPR-based editing of purinergic enzymes, explored preclinically in neuroinflammation and cancer, may eventually enable cell-intrinsic modulation in AS (101–103). Receptor-selective modulators represent another promising frontier. A_2_A receptor agonists suppress inflammation, whereas A_2_B antagonists may limit fibrosis. Despite strong mechanistic rationale, no clinical trials have yet evaluated these compounds in AS. However, P2X7 inhibitors, which target ATP-driven inflammation upstream of adenosine metabolism, have shown benefit in related conditions such as autoimmune uveitis, where they reduce Th17 polarization and tissue damage. These findings suggest that ATP–P2X7 signaling may be a viable target in AS, particularly during early immune activation (38, 84, 104). Combining IL-17 blockade with purinergic modulation could offer synergistic benefit by simultaneously targeting inflammatory and stromal circuits. Because fibroblasts actively reprogram immune cells through cytokine signaling and adenosine metabolism, they should be viewed as active participants rather than passive targets. Therapeutic strategies must therefore address both stromal and immune compartments to disrupt the self-reinforcing loop linking inflammation and ossification (10, 104, 105). Despite their versatility, exosome-based therapies face substantial translational hurdles. Biodistribution remains unpredictable, often leading to hepatic or splenic sequestration. Immunogenicity risks persist, particularly in allogeneic settings, and large-scale manufacturing still lacks standardization and reproducibility. Overcoming these barriers will require precision engineering, rigorous preclinical validation, and improved delivery systems capable of directing exosomes to specific cell populations—particularly immune cells—while sparing fibroblast-rich niches where adenosine may trigger fibrosis (62, 106, 107). We note variable outcomes of ADA inhibition across arthritis models and tissue-specific variability of A_2_B signaling (reports of both pro- and anti-fibrotic effects), underscoring the need for disease- and tissue-context validation before translation to AS.

Adenosine dysregulation in AS shares mechanistic parallels with other autoimmune diseases yet diverges in its structural outcomes. In rheumatoid arthritis, intact CD39 and CD73 expression allows methotrexate to elevate adenosine and suppress TNF-α and IL-6 via A_2_A activation. In systemic lupus erythematosus, elevated ADA activity and variable CD73 expression contribute to immune imbalance, while in psoriatic arthritis, IL-17–driven inflammation may intersect with adenosine signaling, though data remain limited. Comparative analyses are needed to define disease-specific purinergic signatures and determine whether adenosine-targeted therapies should be customized for each autoimmune context (16, 108). Currently, no registered clinical trials directly target adenosine signaling in AS. Ongoing programs focus mainly on TNF-α and IL-17 blockade, leaving the purinergic pathway unexplored. Incorporating purinergic endpoints and exosome-based biomarker analyses into future AS trials could accelerate innovation and clarify therapeutic mechanisms (109). Advancing adenosine-based therapies in AS will require disease-specific validation using integrated experimental systems. Fibroblast–immune co-culture models could delineate cell–cell purinergic interactions, while engineered exosomes with tunable cargo and controlled biodistribution may enable targeted delivery of immunoregulatory enzymes or microRNAs. Biomarker-guided stratification based on CD39/CD73 expression, ADA activity, and receptor profiles could optimize patient selection and dosing. Parallel exploration of receptor-selective modulators and combination regimens with IL-17 blockade may further enhance efficacy (10, 58, 106). Emerging transcriptomic data add another layer of insight. Luan and Wang (2023) identified seven m^6^A RNA-methylation signature genes associated with AS using the GSE73754 dataset. These epigenetic regulators correlated with inflammatory markers and may indirectly influence CD39 and CD73 expression. Incorporating m^6^A profiling into future studies could clarify transcriptional control of adenosine metabolism and refine therapeutic targeting (110).

In summary, restoring adenosine signaling in AS is a promising yet complex endeavor. It demands a network-aware, stage-specific strategy that integrates immune and stromal compartments, leverages exosome-based precision delivery technologies, and prevents fibrosis. Bridging molecular mechanisms with translational innovation will be essential to unlock the therapeutic potential of the purinergic axis in AS.
